# Near-field refractometry of van der Waals crystals

**DOI:** 10.1515/nanoph-2025-0117

**Published:** 2025-05-26

**Authors:** Martin Nørgaard, Torgom Yezekyan, Stefan Rolfs, Christian Frydendahl, N. Asger Mortensen, Vladimir A. Zenin

**Affiliations:** POLIMA – Center for Polariton-driven Light– Matter Interactions, 594932 University of Southern Denmark, Campusvej 55, DK-5230, Odense M, Denmark; Center for Nano Optics, University of Southern Denmark, Campusvej 55, DK-5230, Odense M, Denmark; Department of Physics and Astronomy, Aarhus University, Ny Munkegade 120, DK-8000, Aarhus C, Denmark; Danish Institute for Advanced Study, University of Southern Denmark, Campusvej 55, DK-5230, Odense M, Denmark

**Keywords:** SNOM, vdW crystals, refractive index

## Abstract

Common techniques for measuring refractive indices, such as ellipsometry and goniometry, are ineffective for van der Waals crystal flakes because of their high anisotropy and small, micron-scale, lateral size. To address this, we employ near-field optical microscopy to analyze the guided optical modes within these crystals. By probing these modes in MoS_2_ flakes with subwavelength spatial resolution at a wavelength of 1,570 nm, we determine both the in-plane and out-of-plane permittivity components of MoS_2_ as 16.11 and 6.25, respectively, with a relative uncertainty below 1 %, while overcoming the limitations of traditional methods.

## 1 Introduction

The discovery of the exceptional properties of graphene, enabled by a straightforward exfoliation technique to isolate monocrystalline graphitic films in the early 2000s [1], ignited a search for other materials with layered structures held together by weak van der Waals (vdW) forces. These materials, including transition metal dichalcogenides (TMDCs), have become a focal point in optoelectronics and quantum nanophotonics owing to their unique optical and electrical properties [2], [3], [4], [5]. Due to the layered structure of vdW materials, most of them are expected to exhibit giant anisotropy and even hyperbolic dispersion [6], which cannot be found among previously known naturally occurring materials. TMDCs, for instance, display remarkable optical behavior; their monolayers, readily exfoliated from bulk crystals [7], [8], [9], often exhibit optoelectronic properties distinctly different from those of their bulk counterparts [10], [11]. Molybdenum disulfide (MoS_2_), the TMDC considered in the present work, exemplifies this: it transitions from an indirect bandgap semiconductor with a 1.29 eV bandgap in bulk [12] to a direct bandgap semiconductor with a 1.8–1.9 eV bandgap as a monolayer [13], [14].

Theoretical and computational advancements [11], [15], [16], [17], [18] and experimental explorations [7], [14], [19], [20], [21] have propelled the understanding of vdW materials, yet the experimental characterization of their basic optical properties remains challenging. Exfoliated flakes are generally too small and non-uniform in thickness for traditional refractive index measurements, which limits precision in determining key optical parameters like the complex permittivity tensor. Understanding these optical properties is crucial for the design and optimization of devices based on vdW materials, such as metasurfaces [22], [23], [24], and could enable new applications previously limited by a lack of suitable materials with such unique properties.

Some refractive index measurement methods rely on Snell’s law and goniometer setups to precisely measure refraction angles; however, they are ineffective for slab-like samples, where refraction results only in slight beam displacement. Techniques such as measuring critical angles for total internal reflection or using Brewster’s angle provide rough estimations but lack accuracy due to weak angular dependence. The most advanced method, ellipsometry, measures the change in polarization upon reflection, which is fitted by different models to extract the thickness and refractive index of the unknown layer. However, the precision of conventional spectroscopic ellipsometry relies on using a well-collimated beam with a spot size of >300μm , which is too large to make it reliable for the investigation of exfoliated flakes, because defects and non-uniformities are also illuminated [25], [26]. This limitation is overcome in an imaging ellipsometry setup, which uses angled wide-field illumination and microscopy detection with an objective, claimed to have a lateral resolution below 10 μm [27], [28], [29], [30], [31]. However, its primary purpose is imaging of material contrast, which might compromise the accuracy of refractive index measurements, especially for challenging samples as anisotropic TMDC flakes. Finally, the large refractive index of most TMDC materials results in low sensitivity of the above far-field methods to the out-of-plane component of the refractive index, because the incident illumination from the air even at large angles will be refracted to nearly normal direction inside the flake. One can also retrieve the refractive index by simply measuring the reflection and/or transmission spectra (using either interferometric technique such as Fourier Transform Infrared (FTIR) spectroscopy or by spatially separating different wavelength components using a dispersive prism or a diffraction grating), accompanied by Kramers–Kronig relations if necessary. However, similarly to the ellipsometry, this spectroscopy method is insensitive to the out-of-plane component of the refractive index.

In contrast to far-field methods, scanning near-field microscopy (SNOM) bypasses the issue of lateral resolution. It has been shown that one can use scattering-type (s-)SNOM to obtain quantitative measurements of local dielectric constants by the modification of the scattering strength of the s-SNOM probe by its environment. One approach is based on developing a rigorous model of the probe (a point dipole model, which was later modified into a finite dipole model), which is first applied on known samples for calibration, and then it can be used for measurements [32], [33]. Another approach is based on an empirical search for the probe response function from a set of calibration measurements (black-box calibration) [34], [35]. These methods allow for the extraction of permittivity without detailed electromagnetic modeling of the probe-sample interaction, even accounting for probe tapping effects and far-field background. However, they are developed for isotropic samples, making it challenging to apply them to highly anisotropic vdW materials. Moreover, to our knowledge, they cannot deal with samples where guided modes are excited.

Here we implemented a method we termed ‘near-field refractometry’, which works by probing guided modes within the material, whose properties are directly linked to the material optical properties [36], [37], [38], [39], [40]. Moreover, this approach demonstrated sensitivity to both in-plane and out-of-plane refractive index components, making it uniquely suited for measurements of highly anisotropic vdW materials. It has been shown that the s-SNOM imaging of propagating modes can be used to accompany and refine the far-field refractive index measurements [27], [30], [41]. We show that the optical constants can be precisely determined *alone* from the near-field measurements, when properly done.

**Figure 1:**
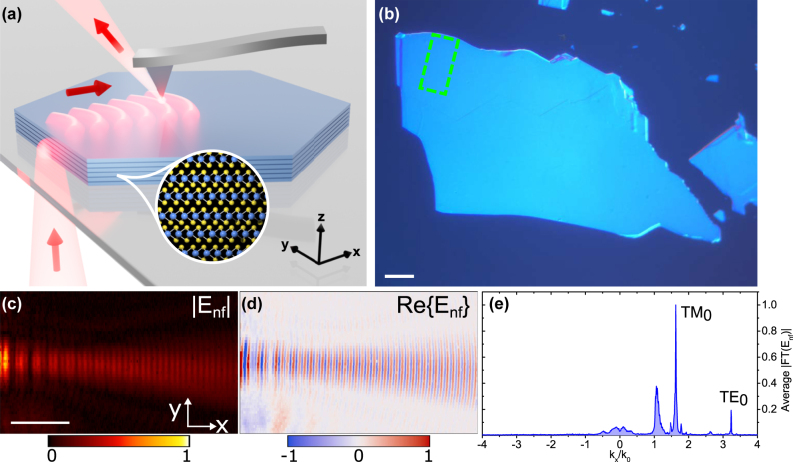
Experimental scheme of the near-field refractometry setup. a) Artistic representation of the experiment where guided modes in an MoS_2_ flake are probed using a transmission s-SNOM setup. b) Differential interference contrast image of a *t* = 185 nm thick MoS_2_ flake. The scale bar is 20 μm. c-d) amplitude $\left|E_{\text{nf}}\right|$ and real part $Re\left\{E_{\text{nf}}\right\}$ of the complex near-field map of the marked 40 μm by 20 μm region in panel b) (green dashed rectangle). The scale bar is 10 μm. e) Momentum-space representation of the complex near-field map, highlighting Fourier components associated with TE_0_ and TM_0_ modes.

We demonstrate the precision and accuracy of our method by investigating a highly anisotropic vdW material, namely MoS_2_ [27], using a phase-resolved SNOM (Figure 1a). We map the near field of guided modes within MoS_2_ flakes of finite thickness (Figure 1c and d) at photon energies below the bandgap (*λ*_0_ = 1,570 nm), with a scan size as small as 40 μm by 20 μm (Figure 1b). First, the guided transverse electric (TE) and transverse magnetic (TM) modes are found by Fourier transforming the recorded near-field map (Figure 1e). After filtering in the Fourier domain, these modes are fitted in the direct space to extract their propagation constant. Finally, after collection of the thickness-dependent dispersion characteristics of these modes, we extract the anisotropic dielectric function of MoS_2_ and estimate its uncertainty.

## 2 Results

To illustrate the general concept of near-field refractometry of vdW materials, we first provide the electrodynamic theoretical foundation, followed by sample fabrication, near-field optical measurements, and data processing to eventually extract the anisotropic optical constants of the vdW material.

**Figure 2:**
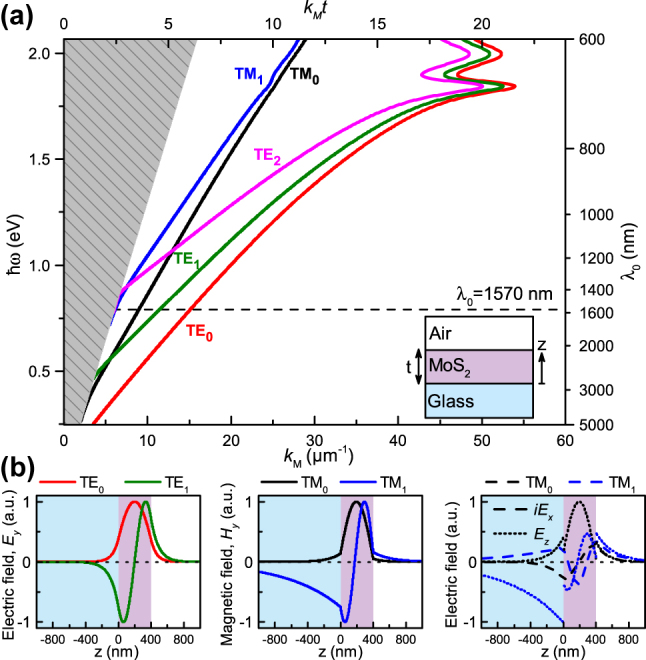
Planar thin-film waveguide modes. a) Dispersion diagram showing photon energy *ℏω* (left vertical axis) and corresponding free-space wavelength *λ*_0_ (right vertical axis) versus mode propagation constant *k*_*M*_ (horizontal axes) for a *t* = 400 nm MoS_2_ flake on BK7 glass substrate for selected TE_*m*_ (*m* = 0, 1, 2) and TM_*m*_ (*m* = 0, 1) modes. The regime of leaky substrate modes is gray shaded, while the horizontal dashed line indicates the experimentally used wavelength. b) Field profiles for the TE_*m*_ and TM_*m*_ modes (*m* = 0, 1), propagating along the *x*-axis.

**Planar thin-film waveguide modes.** We consider a generic anisotropic dispersive dielectric function of a uniaxial vdW crystal flake

(1) $$\varepsilon \left(\omega \right)=\left(\begin{array}{ccc} \varepsilon_{\|}\left(\omega \right) & 0 & 0\\ 0 & \varepsilon_{\|}\left(\omega \right) & 0\\ 0 & 0 & \varepsilon_{⊥}\left(\omega \right) \end{array}\right),$$

where *ɛ*_‖_ and *ɛ*_⊥_ are the in-plane and out-of-plane components, respectively. We assume that the flake of thickness *t* is resting on a substrate with dielectric function *ɛ*_*s*_(*ω*), while being exposed to air above the flake (*ɛ*_*a*_ = 1). Given its high dielectric function (*ɛ* > *ɛ*_*s*_ > *ɛ*_*a*_), the flake is essentially a thin-film waveguide, supporting TE and TM modes, which are guided in-plane of the flake, along the *x*-axis, while being strongly localized in the out-of-plane direction (*z*-direction). The dispersion of TE modes is governed by

(2a) $$q_{\text{TE}}t=atan\left(\frac{k_{a}}{q_{\text{TE}}}\right)+atan\left(\frac{k_{s}}{q_{\text{TE}}}\right)+m\pi ,$$

where $k_{a,s}=\sqrt{k_{M}^{2}-\varepsilon_{a,s}k_{0}^{2}}$ are the confinement factors in the air and the substrate, respectively, and $q_{\text{TE}}=\sqrt{\varepsilon_{\|}k_{0}^{2}-k_{M}^{2}}$ , while *k*_*M*_ is the mode propagation constant, *k*_0_ = *ω*/*c* is the free-space wave vector, and *m* is an integer associated with the mode order. Likewise, the TM modes are governed by

(2b) $$q_{\text{TM}}t=atan\left(\frac{k_{a}\varepsilon_{\|}}{q_{\text{TM}}\varepsilon_{a}}\right)+atan\left(\frac{k_{s}\varepsilon_{\|}}{q_{\text{TM}}\varepsilon_{s}}\right)+m\pi ,$$

where $q_{\text{TM}}=\sqrt{\varepsilon_{\|}/\varepsilon_{⊥}}\sqrt{\varepsilon_{⊥}k_{0}^{2}-k_{M}^{2}}$ . The complex-valued dispersion relation $k_{M}\left(\omega \right)=k_{M}^{\prime }\left(\omega \right)+ik_{M}^{\prime \prime }\left(\omega \right)$ for TE_*m*_ and TM_*m*_ modes of any mode order *m* can be obtained by numerically solving Equations (2a) and (2b) for a given frequency *ω*, flake thickness *t*, and dielectric functions *ɛ*(*ω*) and *ɛ*_*s*_(*ω*). The solution for a *t* = 400 nm thick MoS_2_ flake, supported by a BK7 glass substrate, results in the dispersion diagram illustrated in Figure 2a, where we have used experimentally tabulated dispersive parameters for the MoS_2_ in the Tauc–Lorentz model for *ɛ*(*ω*) [27], while for the BK7 glass, *ɛ*_*s*_(*ω*) is conveniently represented by the Sellmeier dispersion formula. For more details on these representations, see the Supplementary Information (SI) section S1. In the dispersion diagram, the regime of leaky substrate modes $\left(k_{M}<\sqrt{\varepsilon_{s}}\omega /c\right)$ is gray shaded.

At a specific photon energy *ℏω* (or wavelength *λ*_0_ = 2*π*/*k*_0_ = 2*πc*/*ω*), only certain guided modes are supported by the MoS_2_ flake. Adjusting the thickness *t* shifts the guided mode curves along the $k_{M}=\sqrt{\varepsilon_{s}}\omega /c$ line. Consequently, increasing *t* permits more and higher-order modes, while decreasing *t* limits the number of allowed modes in the MoS_2_ flake. In addition to the characteristic thin-film waveguide dispersion relation at low energies, one can also note a clear hybridization with excitons at photon energies around *ℏω* ∼ 1.8–2 eV.

Importantly, SNOM maps the evanescent field in the vicinity of the probe, therefore the mode confinement plays a crucial role in detecting the individual modes. We have calculated the mode profiles at *λ*_0_ = 1,570 nm, which are depicted in Figure 2b. Since s-SNOM is most sensitive to the out-of-plane component of the electric field due to its elongated tip-based scattering probe, the signal of the TM modes will be the strongest, as they contain both *E*_*x*_ and *E*_*z*_ electric field components. In contrast, TE modes only have the *E*_*y*_ field component. More details on the influence of the modes on the detectability is presented in the SI section S2.

Our proposed near-field refractometry method starts with measuring the complex-valued near-field maps of the guided modes, allowing accurate determination of the propagation constant *k*_*M*_(*t*) of each mode, which is then rigorously fitted to the above model, Equations (2a) and (2b), to eventually extract *ɛ*_‖_ and *ɛ*_⊥_.

**Sample fabrication and pre-selection of flakes.** We mechanically exfoliated MoS_2_ flakes with a modified ‘scotch tape’ method (see Methods section). Figure 1b shows a differential interference contrast image of one flake, revealing surface details not clearly visible in the bright- and dark-field images. However, all three complementary imaging methods were used to select flakes with a clean and uniformly thick area, suitable for the precise s-SNOM scanning. Another important aspect of the exfoliation technique used is that as MoS_2_ flakes become thicker, they tend to exhibit more folds and wrinkles, making it harder to obtain large, uniform, flat areas.

It is crucial to have a wide range of flake thicknesses, which should support several guided modes to reliably fit the data to Equations (2a)–(2b) and verify our model, as will be discussed later. For this purpose, we selected six different flakes (labeled from A through F) with the thicknesses varied from ca. 80 to 460 nm, as measured with atomic force microscopy (AFM), each with an estimated uncertainty of ± 10 %.

Furthermore, reflection spectroscopy of each flake can be used to estimate the thickness using the Fresnel equations for anisotropic media [42]; however, this requires *a priori* knowledge of *ɛ*(*ω*) over a wide range of frequencies.

For a detailed view of the MoS_2_ flake characterization, including optical microscopy and reflection spectroscopy, see the SI section S3.

**Near-field measurements.** Our s-SNOM setup can measure both the amplitude and the phase of the evanescent near field, enabling the complete mapping of the complex dispersion relation of guided modes. In our s-SNOM configuration, the light reaches the sample from the opposite side of the scattering tip, which is referred to as transmission-type [36], [37]. Unlike the commonly used reflection configuration [43], this transmission configuration conveniently separates the illumination and detection parts of the setup. This allows mapping the near field of guided modes ‘as launched’ from the edge of the MoS_2_ flakes, without taking the influence of the tip and geometrical decay into account. The concept of transmission s-SNOM is illustrated in Figure 1a, while Figure 1c and d present an example of the obtained near-field map, displayed as the electric near-field amplitude $\left|E_{\text{nf}}\right|$ and its real part Re{*E*_nf_} for the *t* = 185 nm thick MoS_2_ flake. The characteristic fringes in the near-field maps indicate the excitation of more than one guided mode and the interference with the ‘background’ signal. A more detailed schematic of the transmission type s-SNOM is presented in the SI section S4.

In the experiments, a continuous-wave laser with a wavelength of *λ*_0_ = 1,570 nm and a Gaussian beam profile is used as the light source. In this wavelength region, MoS_2_ is expected to have negligible optical loss [27], meaning that the drop in $\left|E_{\text{nf}}\right|$ along the propagation is due to the divergence of the beam itself (Figure 1c). As an alternative to the dispersion diagram, where the frequency is varied (Figure 2a), we instead vary the flake thickness in the experiment. Also, we represent the mode propagation constant in terms of the effective mode index, *N*_*m*_ = *k*_*M*_/*k*_0_, which makes a direct link with the material properties. Essentially, *N*_*m*_ is the weighted average refractive index, experienced by the mode, where the *E*-field of the mode profile is used as the weight. Therefore, the upper limit for *N*_*m*_ is $\sqrt{\varepsilon }$ , when nearly all of the mode *E*-field is concentrated inside flake, whereas $N_{m}\approx \sqrt{\varepsilon_{s}}$ indicates that the mode is weakly confined (close to be leaky substrate mode).

For each scan, we choose an area that encompasses the entire beam along the *y*-axis and is sufficiently large along the *x*-axis to capture enough periods, resulting in a scan size of approximately 40 μm by 20 μm for all measurements. To avoid artifacts (aliasing) and distortion in the complex near-field data, the spatial sampling rate must exceed the Nyquist rate (*f*_*s*_ > 2*f*_max_) for the highest spatial frequency (*f*_max_ = *k*_‖_/2*π*) measured along the propagation direction. To ensure that we resolve all modes for all thicknesses while maintaining consistency, a step size of 25 nm along the propagation direction is chosen for all measurements. Along the *y*-axis, we used a step size of 250 nm.

**Data processing.** To unambiguously extract the ‘pure’ or individual guided modes, the complex near-field maps are filtered to consider only one guided mode at a time. First, by applying a one-dimensional (1D) Fourier transform along the propagation direction (*x* → *k*_*x*_) and averaging along the columns (*y*-axis), we obtain an overview of guided modes supported by the flake. An example of this is shown in Figure 1e, which presents a spectrum containing three prominent peaks. One peak is located close to *k*_*x*_/*k*_0_ ≈ 1, corresponding to the refractive index of free space; this peak we attribute to the diffraction of the incident light from the edge of the flake and other reflections in the system. The second peak, around *k*_*x*_/*k*_0_ ≈ 1.6, is associated with the TM_0_ mode, and is the strongest because of the high sensitivity of s-SNOM to the out-of-plane component of the *E*-field. The third peak, around *k*_*x*_/*k*_0_ ≈ 3.25, corresponds to the TE_0_ mode. In general, these labels can be assigned by solving Equations (2a) and (2b) and using a suitable initial guess for the flake dielectric constant [27]. Alternatively, if optical properties are unknown, the mode assignment can be done by polarizing the normally incident beam along or across the flake edge, which will excite predominantly TE or TM modes, correspondingly. The Fourier spectrum in Figure 1e also shows relatively low signal around *k*_*x*_ ∼ 0, indicating the effectiveness of background removal, while the absence of the signal at *k*_*x*_ < 0 suggests very low back-reflection of the guided modes (*k*_*x*_ → −*k*_*x*_).

One can directly determine the mode propagation constant *k*_*M*_ from the Fourier spectrum, however, the accuracy will be low due to the limited size of the scan. Additionally, since the measured near field does not drop to zero at the edges of the scan, its Fourier spectrum will contain artifacts from the windowing function (also known as apodization function). The commonly used zero padding artificially improves the Fourier resolution, but still plagues the spectrum with artifacts from applying the window, causing spectral leakage of modes in the Fourier domain. To overcome these limitations, we apply extended discrete Fourier transform (EDFT), which essentially iteratively extrapolates the limited-range data to match its Fourier spectrum with the one of the infinite-range data, assuming that its Fourier spectrum is band limited [44]. Finally, to rely less on the parameters of the Fourier transform, we filter each mode in the Fourier domain and fit it in the direct space to determine its propagation constant.

First, we inspect that the field of the filtered mode, *E*_*m*_, follows the expected distribution of the 2D Gaussian beam, propagating along the *x*-axis, which can be written in the following *complex* form [45]:

$$E_{m}\left(x,y\right)=E_{0}\frac{w_{0}}{w}\exp\left(-\frac{y^{2}}{w^{2}}+iN_{m}k_{0}x\right),$$

where $w=w_{0}\sqrt{1+ix/x_{R}}$ is the *complex-valued* diverging beam waist (*w*_0_ ≈ 3 μ*m* is roughly equal to the waist of the incident beam, focused on the flake edge) and $x_{R}=\frac{1}{2}N_{m}k_{0}w_{0}^{2}$ , is the Rayleigh length of the beam. To compensate the divergence, we integrate *E*_*m*_(*x*, *y*) in the *y*-direction, which should result in a pure exponential dependence exp(*iN*_*m*_ *k*_0_ *x*) for the ideal 2D Gaussian beam. Since we don’t expect any absorption losses for the chosen wavelength (meaning *N*_*m*_ is a real number), we find *N*_*m*_ from a slope of the unwrapped phase of *∫E*_*m*_(*x*, *y*) d*y*, with an associated standard error Δ.

**Figure 3:**
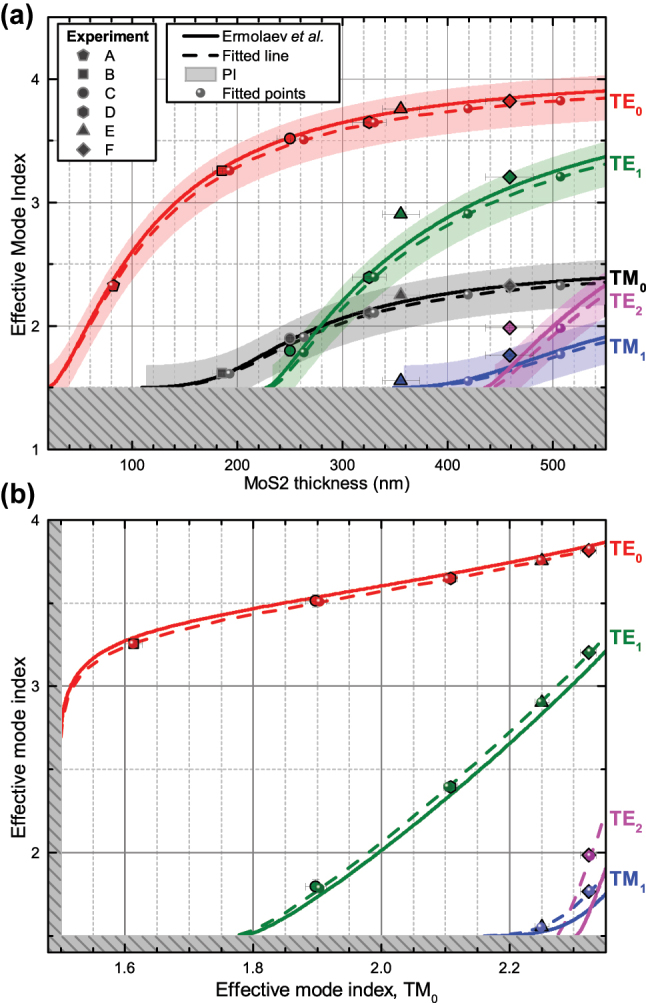
Experimental results and fitting. a) The effective mode index *N*_*m*_ of the different TE_*m*_ (*m* = 0, 1, 2) and TM_*m*_ (*m* = 0, 1) modes as a function of thickness *t* of MoS_2_. b) Parametric plot of the effective mode index of different TE_*m*_ (*m* = 0, 1, 2) and TM_*m*_ (*m* = 1) modes compared to the TM_0_ mode with the thickness *t* varied parametrically. The regimes of leaky substrate modes are gray shaded in both panels. Data points with error bars represent our measurements for flakes A through F, while curves are derived from the dispersion relations using reference permittivities from Ermolaev et al. [27] (solid lines) and our fitted permittivities (dashed lines), which also contain points corresponding to the investigated flakes. Additionally, the shaded areas along the curves in panel a) represent the 95 % prediction interval (PI) for the fitted lines.

The above procedure is repeated for all the measurements for the same flake. Finally, we average *N*_*m*_ within this set using 1/Δ^2^ as a weight, and estimate its uncertainty, accounting for both the error of individual point and the variance of *N*_*m*_ within the set:

(3) $$\Delta N_{\text{eff}}=\sqrt{{avg}_{w}\left(\Delta^{2}\right)+{var}_{w}\left(N_{\text{eff}}\right)},$$

where *w* indicates the same weight 1/Δ^2^. Technical details about the processing of the near-field data can be found in the SI section S5. The obtained effective mode indices for each flake thickness and their associated errors are summarized in Figure 3, which also shows the numerical solutions for each mode corresponding to Equations (2a) and (2b) for varying thickness *t*.

**Experimental data fitting.** Given the effective mode indices for each mode at different thicknesses *t*, it is possible to estimate *ɛ*_‖_ and *ɛ*_⊥_ for MoS_2_ at the used wavelength (here *λ*_0_ = 1,570 nm). The dispersion relations implicitly defines the effective mode indices, which we denote as *N*_*m*_(*ɛ*, *t*) for ease of notation, as functions of *t* and *ɛ* (which includes the two components *ɛ*_‖_ and *ɛ*_⊥_). Therefore, we only treat *t* ^fit^ and *ɛ* ^fit^ as free fitting parameters, and fit our measurements by minimizing the following squared errors

(4) $$\begin{array}{ll} F_{i}\left(\varepsilon^{\text{fit}},t_{i}^{\text{fit}}\right) & ={\left(\frac{t_{i}^{\text{fit}}-t_{i}}{\Delta t_{i}}\right)}^{2}\\ & \;+\sum_{m}{\left(\frac{N_{m}\left(\varepsilon^{\text{fit}},t_{i}^{\text{fit}}\right)-N_{i,m}}{\Delta N_{i,m}}\right)}^{2}, \end{array}$$

where *i* runs over the number of different MoS_2_ flake thicknesses and *m* runs over the different TE and TM modes. The estimated errors of the measurements (Δ*t*_*i*_ and Δ*N*_*i*,*m*_) are used as weights to normalize the discrepancy between measured and fitted *t* and *N*, accordingly. As such, the measurements with a large error will have small influence on the fitting.

One way to perform the fitting is to search for all 8 free fitting parameters at once (two for *ɛ* ^fit^ and six for *t* ^fit^ corresponding to the six different flakes). However, this demands high computational resources and there is a risk of ending in a local minimum. Instead, we used the following nested solver structure. If *ɛ* is known, then the thickness of each flake can be determined separately by minimizing Equation (4) for each flake, which essentially makes implicit definition of $t^{\text{fit}}=t^{\text{fit}}\left(\varepsilon \right)$ . Then this function (involving least-squares solver) is inserted into Equation (4), which is transformed to contain only the free fitting parameter of *ɛ* ^fit^. The latter can easily be found by another least-squares solver.

To find the uncertainty of the determined *ɛ* ^fit^, we apply a perturbation approach, where we modify one of the input parameters (*t*_*i*_ or *N*_*i*,*m*_) and re-iterate the fitting to estimate the sensitivity of *ɛ* ^fit^ to each parameter. We then estimate the uncertainty as the following:

$$\Delta \varepsilon^{\text{fit}}=\sqrt{\underset{i}{\sum }\left[{\left(\frac{\partial \varepsilon^{\text{fit}}}{\partial t_{i}}\Delta t_{i}\right)}^{2}+\underset{m}{\sum }{\left(\frac{\partial \varepsilon^{\text{fit}}}{\partial N_{i,m}}\Delta N_{i,m}\right)}^{2}\right]}.$$

Further details on the fitting procedure are presented in the SI section S6.

Utilizing this method yields a set of fitted thicknesses for each flake, as summarized in Table 1, and the two permittivity components and their associated error, as seen in Table 2.

**Table 1:** Thickness *t* of each MoS_2_ flake measured with AFM (with an estimated uncertainty of ± 10 %), and results from the fitting procedure.

Flake	A	B	C	D	E	F
*t* (nm), AFM	82.4	185.3	250.0	325.3	355.4	458.5
*t* (nm), fitted	81.5	192.7	263.3	330.6	419.3	507.3

**Table 2:** Permittivity components (Eq. 1) of MoS_2_ at *λ*_0_ = 1,570 nm.

	*ɛ*_‖_	*ɛ*_⊥_
Ermolaev et al. [27]	16.56	6.43
Current work	16.11 ± 0.07	6.25 ± 0.04

The effective mode indices, calculated using the experimentally determined permittivities, are presented in Figure 3. These are shown alongside the corresponding curves calculated using permittivity data from Ermolaev et al. [27] for comparison. Overall, our experimental results (Figure 3 and Table 2) show qualitative agreement with previously reported data [26], [27]. However, at a quantitative level, the results reveal a difference in the effective mode index, most notably for the TE modes. One should also note that there is a significant difference between the thicknesses measured with AFM and the fitted ones (Table 1), with the discrepancy increasing as the thickness becomes larger. This can partially be attributed to the calibration being done with a calibration grating of 100 nm high steps, meaning that the thicker flakes may be outside of the calibration region and the scanner is non-linear for those step heights. However, the discrepancy is uncommonly high and non-monotonic, suggesting other causes yet to be found.

By representing the data in a parametric plot, where the effective mode indices are compared to the one of TM_0_ mode (for thicknesses where they co-exist), we eliminate the uncertainties associated with specific values of *t* (Figure 3b). This plot clearly shows the difference between our measurements and calculations using the reported optical constants [27]. Moreover, the localization of our measured points close to the fitted lines suggests high accuracy of our method. This demonstrates that the used method is both suitable and capable of determining the permittivity of crystalline TMDC flakes with lateral dimensions as small as tens of microns, which are otherwise challenging to probe using standard ellipsometry techniques due to the limited surface uniformity over larger flake areas and spot size.

## 3 Discussion

This work demonstrates a novel method for determining the optical properties of materials by using transmission-type s-SNOM to measure the real part of its permittivity. By measuring the complex near-field map of mechanically exfoliated MoS_2_ flakes of varying thicknesses on BK7 glass, we determined the permittivity components of MoS_2_ with a relative error of ∼0.5% , while probing only areas of approximately 40 μm × 20 μm. At the first glance, the estimated error is much smaller than the one expected from the Fourier transform (for our measurements with 40-μm-long range, the resolution of Fourier spectrum is Δ*k*/*k*_0_ ≈ 0.04, which is more than 1 % of *N*_*m*_ for all studied guided modes). However, the uncertainty in determining the position of the peak (which can be found, for example, by locally fitting with a Lorentzian function) can easily be much smaller than the Fourier resolution, when it is known that it should be a peak of a single mode, and the signal-to-noise level is high enough. Similarly, the frequency of a sine curve can be accurately determined by fitting, even if the interval is less than the sine period itself (i.e., when the resolution of the Fourier spectrum is less than the frequency itself). In fact, each fitting of the guided mode in our measurements resulted in the error of Δ ∼ 0.001. However, the variations between measurements with different flake orientations resulted in the error of Δ ∼ 0.01, according to Equation (3). We attribute this variation to the imperfect synchronization of the bottom parabolic mirror during the scan (see Methods), which can be improved to further increase the accuracy of determining the permittivity. Alternatively, these errors indicate that the probing area can be reduced in the current setup without compromising the accuracy of the method.

As a proof of concept, we focused on a known low optical loss region of MoS_2_ and therefore only analyzed the real part of the effective mode indices, which is associated with the real part of the permittivity. By analyzing the decay in amplitude of $\left|E_{\text{nf}}\right|$ along the propagation direction, it is possible to determine the imaginary part as well (when the divergence of the guided Gaussian beam is taken into account). However, in frequency regions where the material exhibits high optical losses, the field will decay rapidly, limiting the extent of the detectable region and thus the precision of the results. In particular, the dispersive coupling to other interactions, such as excitons seen in Figure 2a, would also influence the decay. For sufficiently thick flakes, the near-field signal also decreases in overall strength for some modes, as most of the field becomes confined within the flake itself, making near-field measurements more challenging.

Our results show a slight difference in the effective mode indices of the TE and TM modes compared to the expected values based on literature permittivity values [26], [27], indicating a discrepancy in the in-plane (*ɛ*_‖_) and out-of-plane (*ɛ*_⊥_) permittivity components. The discrepancy in *ɛ*_⊥_, found to be ∼3 % from the ellipsometry data reported by Ermolaev et al. [27], can be ascribed to s-SNOM’s high sensitivity to out-of-plane polarized fields. In contrast, ellipsometry of high-index materials suffers from small refracted angles, resulting in small out-of-plane *E*-field components and correspondingly low sensitivity to *ɛ*_⊥_. Surprisingly, we have also found a difference of ∼3 % in *ɛ*_‖_, where ellipsometry is supposed to provide accurate measurements.

In the proposed method, measurements are restricted to a single wavelength at a time, which limits its applicability for broadband determination of optical properties. To overcome this limitation, one could use a broadband source and another detection scheme, known as nano Fourier-transform infrared spectroscopy (nano-FTIR), where one records a full interferogram at each point and consequently applies the Fourier transform to transform it to a spectrum [46]. However, this would significantly increase the acquisition time for a similarly sized scan area; therefore, there may consequently be a need to decrease the number of lateral sampling points.

While we have illustrated the principle with measurements on MoS_2_, we emphasize its applicability to the broader class of vdW materials, including uni- and bi-axial semiconductors [26], [29], [41], [47], [48], [49], metals [50], semimetals [51], [52], topological insulators [53], [54], [55], [56], and even non-vdW materials [57]. Additionally, one can modify guided modes by using a different substrate, which might improve the sensitivity. For example, by having a metallic mirror as a substrate, vdW flakes will support plasmon polaritons [39], [58] and image polaritons at longer wavelengths [59], [60], [61]. The essential part of our technique, to measure anisotropic properties, is that the investigated sample supports few diverse guided modes, featuring both in-plane and out-of-plane *E*-field components. Having two or more modes per flake allows one to determine its thickness from fitting instead of relying on mechanical measurements (for example, by AFM), which appeared to be imprecise. The precision of our method is directly linked to the length of the near-field map (due to the properties of the Fourier transform), therefore we expect lower precision for lossy modes (due to the leakage or absorption losses). Finally, the accuracy of our method depends on how much the mode propagation properties depend on the optical properties of the investigated material, therefore it will be less accurate in determining R{ε}≪−1 of metal-like materials, since the effective mode index of the supported surface plasmon polariton mode is close to the refractive index of the neighboring material. However, it is still precise in determining I{ε} from mode propagation length [37].

In conclusion, the proposed method for determining the permittivity of materials using transmission-type s-SNOM serves as an addition to the well-studied ellipsometry method, specifically, for accurate optical characterization at the microscale.

## 4 Methods

**Substrate cleaning procedure.** The SCHOTT N-BK7^®^ glass substrates are ultrasonicated in acetone followed by isopropyl alcohol (IPA) for 5 min each. Acetone can dissolve non-polar and polar compounds (like oil and organic compounds), however acetone leaves residues. IPA removes the remaining acetone and can also dissolve non-polar compounds. The ultrasonication loosens particles and residues adhering to the surface. As a last step the substrates are rinsed with deionized water and dried with pressurized nitrogen (N_2_).

**Mechanical Exfoliation procedure.** To mechanically exfoliate MoS_2_, residual free wafer-tape (Nitto Denko Corporation) is used. The ‘mother’ crystal is placed in contact with the tape, and when it is pulled away, a significant amount of material is transferred to the tape. By repeatedly sticking and unsticking the tape to itself in the area with the material, the crystals gradually become thinner. Afterwards, a polydimethylsiloxane (PDMS) stamp (Gel-Pak 8) is brought into contact with a selected area of the tape and then peeled off, leaving flakes on the PDMS. The PDMS is then placed on a glass slide with the flake facing away from the glass surface. Using a manipulation stage, the glass slide and PDMS are slowly lowered toward a BK7 glass wafer chip mounted on a vacuum chuck at 60 °C, where they eventually make contact (the procedure is monitored through an optical microscope). Lastly, the PDMS stamp is slowly lifted up, leaving MoS_2_ flakes on the glass chip.

**Near-field setup.** The near-field measurements were performed using a customized commercially available transmission type s-SNOM (NeaSpec, Attocube), and a sketch of the setup is given in the SI section S3. The setup employs pseudo-heterodyne demodulation to simultaneously acquire amplitude and relative phase information from the near-field signal. A continuous-wave near-infrared laser beam (*λ*_0_ = 1,570 nm) is split into two paths. One path is the reference arm, where the light is modulated by an oscillating mirror (*f* ≈ 300 Hz). In the other path the laser beam is focused ( ∼3μm spot size) onto the edge of a flake by a parabolic mirror (PM) below the sample. A near-field probe (Pt-coated ARROW-NCPt, NanoWorld) scatters the near field into free space, transforming the bound evanescent waves into freely propagating waves.

The scattered near-field signal is collected by another PM above the sample and is further recombined with reference beam so their interference can be detected. The detected signal is then subsequently demodulated (pseudo-heterodyne detection) at higher harmonics of the probes oscillation frequency (*η*Ω, with *η* = 3 and 4) to suppress the background (any light scattered from the tip or the sample, but not related to the probed near field).

When scanning, the sample is moved and to maintain the excitation beam spot at the flake edge, the bottom PM is moved synchronously with the sample. However, the stage for the bottom PM is not as precise as the sample stage, which mainly results in a small artificial phase ‘wobbling’ of the excitation light. This leads to spectral leakage in the Fourier domain, which can be seen as small sidebands around guided modes (see, for example, TM_0_ mode in Figure 1e). This, in turn, can result in the incorrect determination of *N*_*m*_ for closely spaced modes, when their spectral leakage will overlap (for example, TM_1_ and TE_2_ for the 460-nm-thick flake). To correct the phase ‘wobbling’ and determine *N*_*m*_ without artificially lowering the estimated errors, we use the following two-step procedure:

*i)* First, we select the mode with the most prominent peak in the Fourier spectrum, which does not overlap with others, and filter it using a square window function with a width of 0.5*k*_0_. Then it is inversely transformed back to real space, converted to 1D by performing integration along the *y*-direction, followed by a linear fit of the unwrapped phase. This process provides the residual phase, which is then subtracted from the raw data.

*ii)* In the second iteration, the corrected complex near-field data is Fourier transformed again and filtered for each mode using a smaller square window function with a width of 0.15*k*_0_, followed by the same procedures to provide fitted *N*_*m*_. Importantly, to avoid artificial lowering of the uncertainty, we estimate the squared error of *N*_*m*_ for each mode as the sum of the squared error in the second step and the squared error for the reference mode in the first step.

## Supplementary Material

Supplementary Material Details
