# Supplementary material for: Near-field refractometry of van der Waals crystals
Source: Nanophotonics. 2025 May 26;14(14):2473–83. doi: 10.1515/nanoph-2025-0117 (PMC12273539; doi:10.1515/nanoph-2025-0117)
Supplement: Supplementary file 1 — Supplementary Material Details [file j_nanoph-2025-0117_suppl_001.pdf]

Supplementary Information

S1. Material permittivities

The relative permittivity of the BK7 glass substrates are given by the Sellmeier dispersion formula

$$\varepsilon_s(\omega) = 1 + \sum_i \frac{B_{s,i}\lambda^2}{\lambda^2 - C_{s,i}}, \quad \lambda = 2\pi c/\omega, \tag{S1}$$

where $B_{s,i}$ and $C_{s,i}$ are experimentally determined Sellmeier coefficients. The coefficients for BK7 glass are presented in Table S1.

$B_{s,1}$	$B_{s,2}$	$B_{s,3}$
1.039612120	0.231792344	1.010469450
$C_{s,1}$ (μm^2)	$C_{s,2}$ (μm^2)	$C_{s,3}$ (μm^2)
0.006000699	0.0200179144	103.56065300

Table S1. Sellmeier coefficients for BK7 glass [1].

The relative permittivity of MoS_2 used to find the modes in the dispersion diagram (Figure 2 in the main text) is from Ermolaev et. al. [2] where they experimentally determined it using imaging ellipsometry and the Tauc–Lorentz oscillator model. The in-plane relative permittivity is determined by

$$\varepsilon_{\|}(E) = \varepsilon_\infty + \varepsilon_{\text{uv}}(E) + \sum_i \varepsilon_{\text{TL},i}(E), \tag{S2}$$

where E is the photon energy ($E = \hbar\omega$), ε_∞ is the relative permittivity at infinite photon energy, $\varepsilon_{\text{TL},i}(E)$ is the complex Tauc–Lorentz oscillator function, and ε_{uv} is the added ultra-violet (UV) pole:

$$\varepsilon_{\text{uv}}(E) = \frac{A_{\text{uv}}}{E_{\text{uv}}^2 - E^2}, \tag{S3}$$

with the amplitude $A_{\text{uv}} = 228\,\text{eV}^2$ and energy position $E_{\text{uv}} = 15\,\text{eV}$, accounting for strong absorption in the UV.

The Tauc–Lorentz oscillator model for the imaginary part of the in-plane permittivity is related to its real part through the Kramers–Kronig transform

$$\text{Im}\left[\varepsilon_{\text{TL},i}(E)\right] = \begin{cases} \frac{A_i E_{0,i}\Gamma_i (E-E_{g,i})^2}{(E^2-E_{0,i}^2)^2+\Gamma_i^2 E^2} \cdot \frac{1}{E}, & E > E_{g,i} \\ 0, & E \le E_{g,i} \end{cases}, \tag{S4}$$

$$\text{Re}\left[\varepsilon_{\text{TL},i}(E)\right] = \frac{2}{\pi}\int_{E_g}^{\infty} \frac{\xi\,\text{Im}\left[\varepsilon_{\text{tl},i}(E)\right]}{\xi^2 - E^2}\text{d}\xi, \tag{S5}$$

where A is the oscillator (exciton) strength (amplitude) of the peak, Γ is the broadening term of the peak, E_g is the material's optical bandgap energy, and E_0 is the peak central energy. The values used are collected in Table S2.

i	A (eV)	E_0 (eV)	Γ (eV)	E_g (eV)
1	308	1.852	0.067	1.761
2	135	2.006	0.148	1.82
3	19.3	2.662	0.380	1.24
4	69	2.99	1.348	1.31

Table S2. Tauc–Lorentz oscillator model parameters provided by Ermolaev et al. [2].

For the out-of-plane component we use Cauchy's equation with two terms

$$\text{Re}\left[\varepsilon_\perp(\lambda)\right] = \left(A_c + \frac{B_c}{\lambda^2}\right)^2, \tag{S6}$$

$$\text{Im}\left[\varepsilon_\perp(\lambda)\right] = 0, \tag{S7}$$

where $A_c = 2.463$ is the refractive index at infinite wavelength and the coefficient $B_c = 119 \cdot 10^3(\text{nm}^2)$ accounts for the first-order wavelength dependency. The resulting permittivities in the form of refractive index (n) and extinction coefficient (κ), $\varepsilon = (n + i\kappa)^2$, are shown in Figure S1, together with two other measurements from the literature.

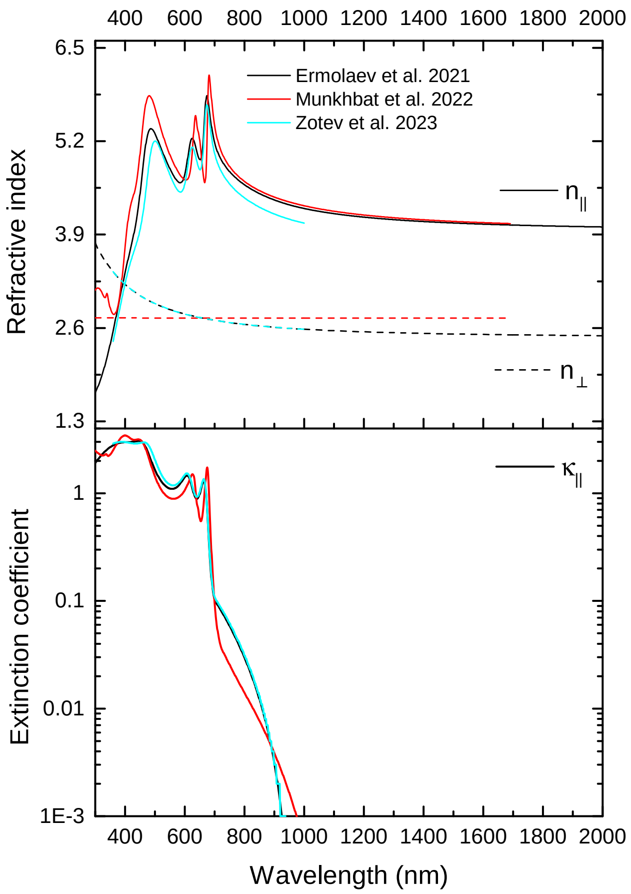

FIG. S1. Refractive index (n) and extinction coefficient (κ) of MoS_2 from Ermolaev et al. [2], Munkhbat et al. [3], and Zotev et al. [4]. There is a notable difference between measured refractive indices even in the low-loss region (λ > 700 nm). Imaging ellipsometry was used in [2] and [4], while a conventional ellipsometry setup was used in [3].

S2. SNOM probing sensitivity to different modes

The field distribution of the field components for the first two orders of the TE and TM modes for each thickness is depicted in Figure S2 as a color gradient.

The mode distribution significantly influences the detectability of modes when using s-SNOM. The scattered signal in s-SNOM is dependent on the orientation of the electric field components relative to the tip, with the strongest signal occurring when the components are parallel. This is because the interaction between the tip and the electric field is maximized when they are aligned. For TE modes, the electric field has a single in-plane component (E_y), which substantially reduces the scattering and collection efficiency. Conversely, for TM modes, the electric field components include both in-plane (E_x) and out-of-plane (E_z) elements, resulting in relatively higher scattering efficiency.

The detectability of modes is also influenced by the mode order and waveguide thickness. Higher-order modes, with more complex field distributions, typically exhibit weaker confinement and lower detectability compared to fundamental modes. Thinner waveguides enhance the interaction between the extended electromagnetic field and the s-SNOM tip, improving detectability. However, as the waveguide thickness increases, a larger portion of the electromagnetic field is confined within the waveguide, reducing the field's interaction with the tip and thus decreasing detectability. These effects are crucial for accurately interpreting results.

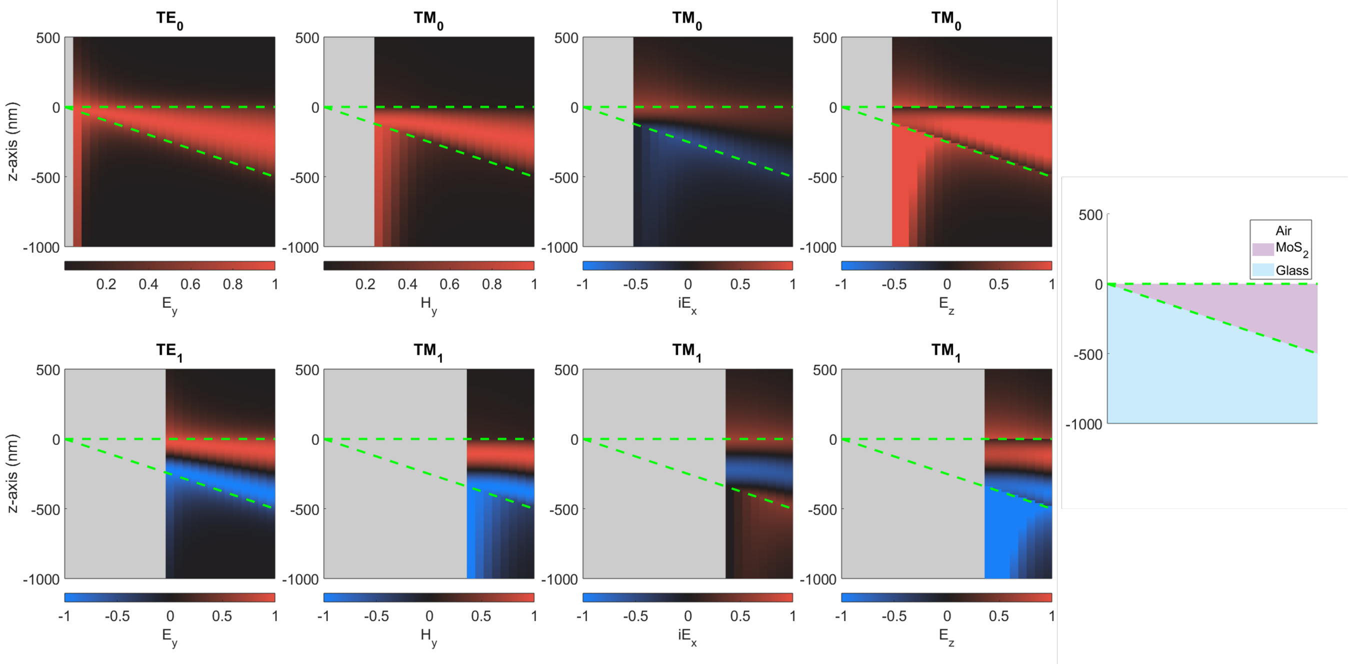

FIG. S2. Field distributions for TE_m (m=0,1) and TM_m (m=0,1) modes for varying thickness of MoS_2. The gray area is the region of leaky modes. The field of TE modes are normalized by the maximum of E_y for each thickness. Similarly, TM modes are normalized by the maximum of H_y for each thickness. Thus, one can directly compare E_x and E_z fields in strength and verify the dominance of the normal E_z component for TM modes.

S3. MoS_2 flake characterization

Imaging of flakes

The mechanically exfoliated MoS_2 flakes on BK7 glass are characterized by conventional optical microscopy using a optical microscope (Axiotech, Zeiss) which allows for bright-field (BF), dark-field (DF) and differential interference contrast (DIC) imaging. The images of the measured flakes are presented in Figure S3.

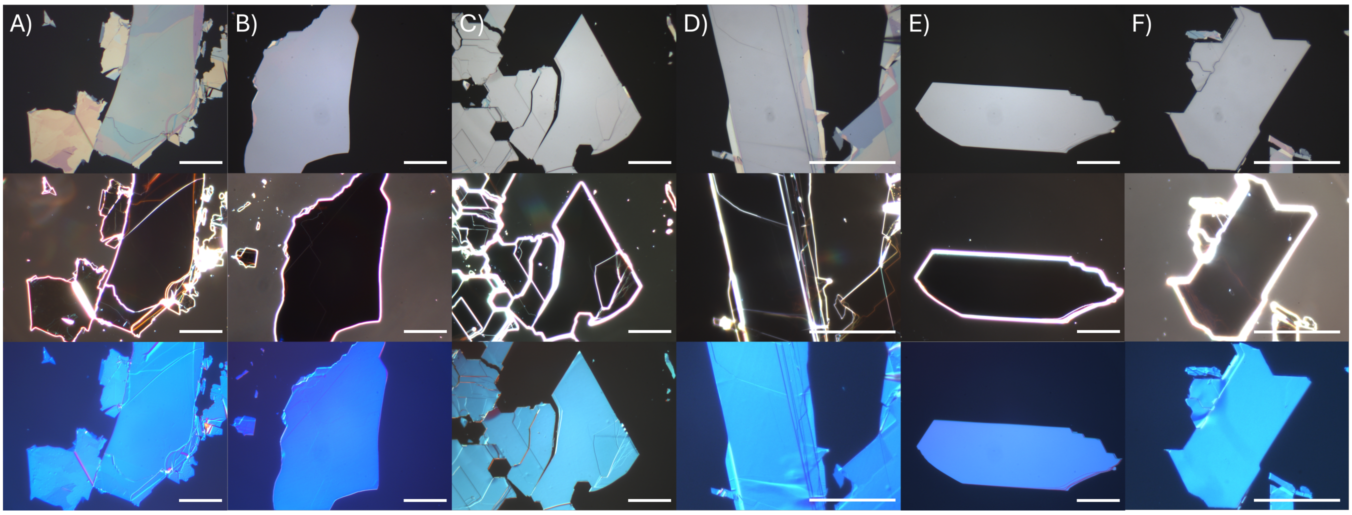

FIG. S3. Bright-, dark-field, and DIC images of the flakes measured. From **A)** to **F)** the thicknesses are as measured by AFM given in Table S3. The scale bars are 50 μm.

AFM measurements of flake thickness

The thickness of each flake is determined using AFM by scanning over the flake edge (the edge is parallel to the slow scan y-axis) in several slightly different locations. The raw data from each scan is corrected for tilt and offset (line shift) as the following:

$$z(x, y) = z_{\mathrm{raw}}(x, y) - ax - b(y), \tag{S8}$$

where slope a and offset $b(y)$ are found by minimizing $\sum z^2$ for points corresponding to the substrate. The resulting thicknesses for each flake are collected in Table S3.

Flake	A	B	C	D	E	F
Thickness (nm)	82.4	185.3	250	325.3	355.4	458.5

Table S3. AFM measured thicknesses.

Raman spectroscopy

To verify that the flakes are made of MoS_2 (HQ-graphene, 2H-MoS_2 natural crystal) we use Raman spectroscopy to compare the peaks in the Raman shift to values given in the literature. Figure S4 shows the region where the in-plane E^1_{2g} and out-of-plane A_{1g} Raman modes are active.

Bulk MoS_2 exists in different crystalline phases (the stacking of the individual layers is different): 1T (octahedral symmetry), 2H (hexagonal symmetry), and 3R (rhombohedral symmetry). The Raman spectrum permits us to distinguish between these since the 1T-phase has Raman modes that are different from the two other phases. The 2H and 3R phases have peaks at $\sim E^1_{2g} = 385\,\mathrm{cm}^{-1}$ and $\sim A_{1g} = 410\,\mathrm{cm}^{-1}$ for the in-plane and out-of-plane vibrational modes respectively (with a difference between the two peaks

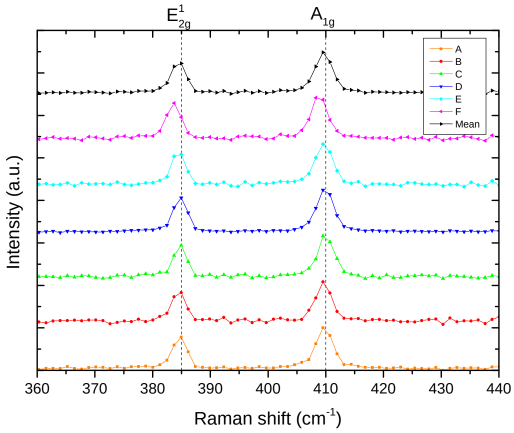

FIG. S4. Raman spectra of the individual flakes on BK7-glass and their MoS_2 Raman-modes using a laser with a (non-excitation) wavelength of 532 nm. Literature values (vertical dashed lines) for Raman-modes of MoS_2 are taken from Carvalho et al. [5].

of ~ 25 cm^{-1}) [5]. For the 1T-phase the in-plane E_{2g} mode is absent and out-of-plane ~ A_{1g} = 405 cm^{-1} [6]. To distinguish the 3R phase from the 2H phase one can look at the second harmonic generation that is present for bulk 3R-MoS_2 because of it being non-centrosymmetric (lacks inversion symmetry).

MoS_2 show different optical properties depending on the different crystalline phases and its thickness when it approaches a few layers (each layer is ~ 0.65 nm in thickness). However, we only considered bulk flakes and can thus neglect the thickness dependent intrinsic optical material properties.

Thickness measurements by reflection spectroscopy

Another way to characterize the MoS_2 flakes is with reflection spectroscopy, where it's possible to extract the thickness of the flakes assuming the permittivity is known (or vice versa). To this end we need the Fresnel equations for the case of a uniaxial crystal. A sketch of the configuration is shown in Figure S5.

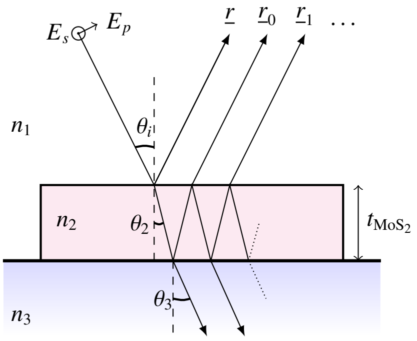

FIG. S5. A plane wave incident on a thin dielectric layer of thickness t on a semi-infinite thick substrate.

Generally, the refractive indices, $n_i(\omega)$, vary with frequency. In a uniaxial crystal, the refractive index encountered by a p-polarized plane wave propagating within the crystal is influenced by the refracted angle θ_2, which in turn depends on the incidence angle θ_i and the refractive indices of the first layer and both components of the second layer. In contrast, for an s-polarized plane wave, the electric field interacts solely with the in-plane components of the layers. These factors, along with the Fresnel equations for the complex amplitude reflection coefficients for s- and p-polarized light at the two interfaces, are expressed as follows:

s-polarization equations

$$n_2 = n_{\|},$$

$$\sin(\theta_2) = \frac{n_1}{n_{\|}} \sin(\theta_i),$$

$$r_{s,12} = \frac{n_1 \cos(\theta_i) - n_2 \cos(\theta_2)}{n_1 \cos(\theta_i) + n_2 \cos(\theta_2)},$$

$$r_{s,23} = \frac{n_2 \cos(\theta_2) - n_3 \cos(\theta_3)}{n_2 \cos(\theta_2 + n_3 \cos(\theta_3)},$$

p-polarization equations

$$n_2 = \left(\frac{\cos^2(\theta_2)}{n_{\|}^2} + \frac{\sin^2(\theta_2)}{n_{\perp}^2} \right)^{-\frac{1}{2}},$$

$$\sin(\theta_2) = \left(1 + \frac{n_{\|}^2}{n_1^2 \sin^2(\theta_i)} - \frac{n_{\|}^2}{n_{\perp}^2} \right)^{-\frac{1}{2}},$$

$$r_{p,12} = \frac{n_1 \cos(\theta_2) - n_2 \cos(\theta_i)}{n_1 \cos(\theta_2) + n_2 \cos(\theta_i)},$$

$$r_{p,23} = \frac{n_2 \cos(\theta_3) - n_3 \cos(\theta_2)}{n_2 \cos(\theta_3) + n_3 \cos(\theta_2)},$$

where the subscript numbers indicate the mediums of the interfaces (e.g. $r_{s,12}$ is the reflection from the interface between medium 1 and 2 where the light comes from medium 1). The light gets refracted and reflected from each interface in a series of events and gives the complex reflection coefficients:

$$\begin{aligned} \underline{r} &= r_{12}, \\ \underline{r}_0 &= t_{12} r_{23} t_{21} \mathrm{e}^{2i\beta}, \\ \underline{r}_1 &= t_{12} r_{23} t_{21} \mathrm{e}^{2i\beta} r_{21} r_{23} \mathrm{e}^{2i\beta}, \\ &\vdots \\ \underline{r}_m &= t_{12} r_{23} t_{21} \mathrm{e}^{2i\beta} \left(r_{21} r_{23} \mathrm{e}^{2i\beta} \right)^m, \end{aligned} \tag{S9}$$

where the total sum ($\underline{r}_{tot} = \underline{r} + \underline{r}_0 + \underline{r}_1 + \ldots$) is a geometric series that converges to

$$\underline{r}_{tot} = \frac{r_{12} + r_{23} \mathrm{e}^{2i\beta}}{1 + r_{12} r_{23} \mathrm{e}^{2i\beta}}, \quad \beta = \frac{2\pi n_2 t_{\mathrm{MoS_2}} \cos(\theta_2)}{\lambda_0}, \tag{S10}$$

for *s*- and *p*-polarized light using the appropriate coefficients from above. The reflectance is given as $R = \left| \underline{r}_{tot} \right|^2$ and for unpolarized light one can use, $R = \left| \underline{r}_{s,tot} \right|^2 /2 + \left| \underline{r}_{p,tot} \right|^2 /2$, instead. The total reflectivity can also be found by integrating the reflectance over all the angles ($0 \rightarrow \pi/2$) as in the following:

$$R_{NA} = \int_0^{\pi/2} R(\theta)\, F(\theta)\, d\theta, \tag{S11}$$

where $F(\theta)$ is used to take into account the numerical aperture (NA) of the objective. We assume $F(\theta)$ to have a Gaussian distribution of

$$F(\theta) = \frac{2}{\sqrt{2\pi\theta_i^2}} \exp\left[-\left(\frac{\theta}{\theta_i} \right)^2 \frac{1}{2} \right], \tag{S12}$$

where θ_i corresponds to the collection half-angle of the objective:

$$\theta_i = \sin^{-1}\left(\frac{\mathrm{NA}}{n_1} \right). \tag{S13}$$

In order to see the influence of the incident angle, we compare reflectance spectra for *s*- and *p*-polarized light at normal incidence and at the angle, corresponding to the collection half-angle of our objective (Figure S6). As expected, inclined incidence results in a blue shift of the resonances, which is smaller for the *s*-polarization (partially because of the corresponding larger refractive index of the flake and, thus, smaller refracted angle θ_2). When we take into account the collection by the objective and integrate the reflectance, then the resultant spectrum features a moderate blue shift. The dependence of the resonance blue shift on the incident angle and corresponding NA for the integral form of R_{NA} is illustrated in Figure S6b.

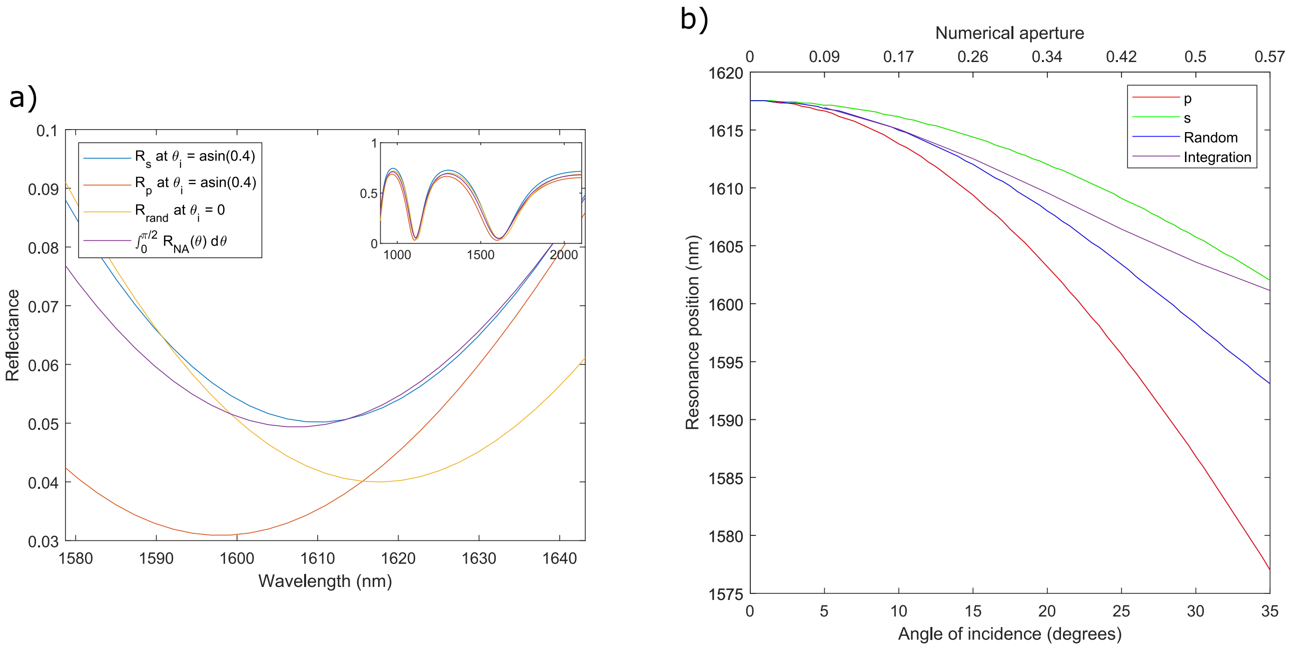

FIG. S6. **a)** Reflectance spectra in a spectral region around a resonance, calculated for normal and tilted incidence for *s*- and *p*-polarized light, compared with integrated un-polarized reflectance. The insert shows the reflectance over the entire wavelength region considered in the measurements. **b)** The influence of the incident angle (and corresponding NA for the integral form of R_{NA}) on the blue shift of the resonance.

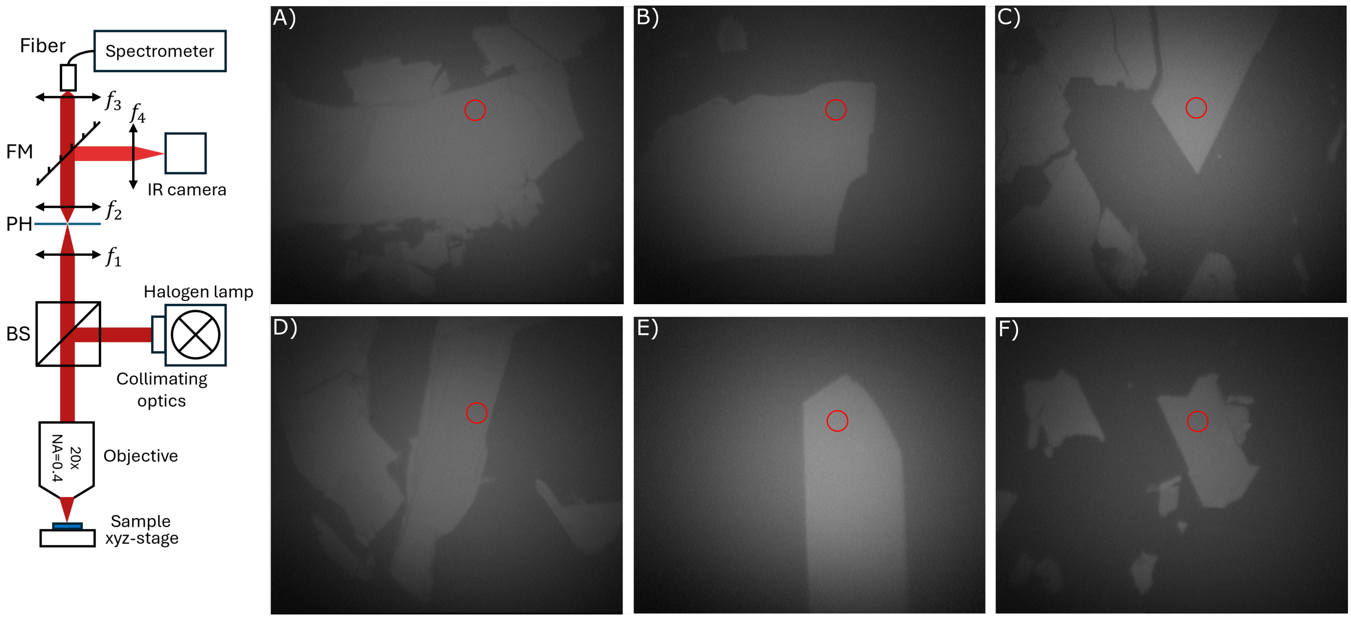

FIG. S7. Left, spectroscopy set-up, with the focal length of the lenses f_1 = 20 cm, f_2 = 10 cm, f_3 = 3 cm, and f_4 = 20 cm. The abbreviations are beam-splitter (BM), 300 µm pinhole (PH), and flip-mount mirror (FM). The pinhole is used to spatially filter the area of the sample, from which the spectrum is collected, and it roughly corresponds to a circle of 15 µm in diameter (because of 20× magnification). Right, IR images of flakes A-F with red circles marking the sample area, where the reflection spectra were collected.

In the experiment, we used a halogen lamp as the light source, a near-infra-red spectrometer (NIRQUEST, Ocean Optics, grating NIR2 900-2200 nm, slit 50 µm), and a NA = 0.4 objective (LMPlan IR 20×/0.40) together with various optical components to obtain the spectra. The setup and the measured area of each flake are shown in Figure S7.

The measured reflectance spectra from each flake were normalized by the reflectance from a gold mirror (Thorlabs), preceded by the subtraction of the dark counts background (Figure S8). Usually optical spectra are used to find the position

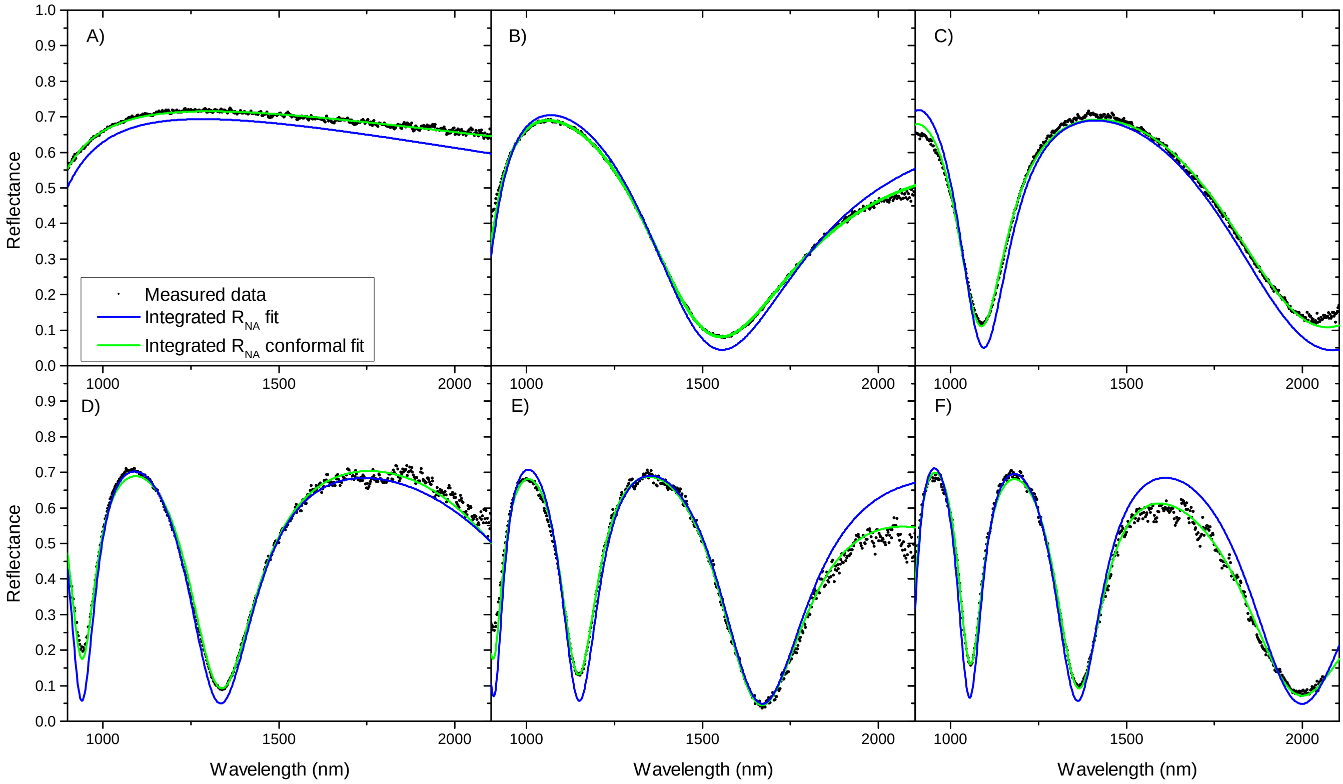

FIG. S8. Reflection spectroscopy of the different samples. The assumed refractive index is from Ermolaev et al. [2]. The conformal fits are done with the following fixed parameters: **A)** $p_{2,3,5,6} = 0$, **B)** $p_{2,3,6} = 0$, **C)** $p_{3,6} = 0$, **D)** $p_{3,6} = 0$, **E)** $p_3 = 0$, and **F)** no fixed parameters.

of minima/maxima, because small misalignments, scattering, imperfections, instability of the source spectra, or inconsistent background in the system causes the absolute values to be slightly higher or lower than expected by theory. To overcome this and use all of the spectral data (data points below 920 nm and above 2050 nm are excluded in the fitting due to low signal to noise ratio), we introduce a few extra fitting parameters to conformally transform the spectra reflectance-axis. Essentially, we would like to transform both the bottom ($R(\lambda) = 0$) and the top ($R(\lambda) = 1$) into slowly varying function $f(\lambda)$, which we selected as following:

$$f(\lambda) = p_i + p_j \sin(\pi\lambda_{\text{norm}}/10 + p_k), \tag{S14}$$

where $p_{i,j,k}$ are free fitting parameters and

$$\lambda_{\text{norm}} = \frac{\lambda - \lambda_{\min}}{\lambda_{\max} - \lambda_{\min}} - 0.5, \tag{S15}$$

is the normalized wavelength. As one can see, this definition ensures slow variation of $f(\lambda)$. This function allows the flexibility in terms of what transformation is needed: a simple offset can be defined by fixing $p_j = 0$ and $p_k = 0$, while tilt is achieved by fixing $p_k = 0$. Therefore, we transform our theoretical spectra into

$$\begin{aligned} M(\lambda) &= f_1(\lambda) + R_{NA} f_2(\lambda) \\ &= p_1 + p_2 \sin(\pi\lambda_{\text{norm}}/10 + p_3) + R_{NA}(\lambda)\left[1 + p_4 + p_5 \sin(\pi\lambda_{\text{norm}}/10 + p_6)\right], \end{aligned} \tag{S16}$$

where p_{1-6} are free fitting parameters, which is then used to fit our experimental spectra (Figure S8). The form of the conformal transformation was selected to rescale the reflectivity curve without severely changing the position of its extrema, which can be confirmed in a detailed Figure S9.

The results of both the fit with $R_{NA}(\lambda)$ and the conformal fit with $M(\lambda)$ are displayed in Table S4. As one can see, the fitting results of the near-field measurements coincide much better with the ones of fitting the reflectance spectra, compared to AFM measurements (especially for thick flakes E and F). The introduction of the conformal transform for fitting the reflection spectra has a tiny influence on the fitted thickness, but it reduces the estimated error due to a better match between the measured points and fitted curve. It should be kept in mind, however, that the fitting of the reflection spectra relies on *a priori* knowledge of the

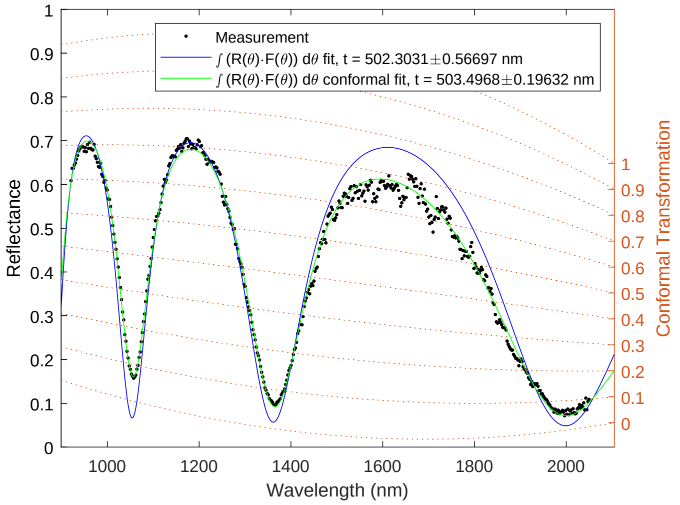

FIG. S9. Influence of the conformal transformation. The measured reflectance of flake F (dots), fitted with the integrated reflectivity R_{NA} (solid blue line) and with the conformally transformed R_{NA} (solid green line). The conformal transformation of different reflectance levels are depicted in the right orange scale. As such, the conformal fit does not result in significantly different fitted thickness, but it adjusts the fitted curve to align more closely with the measured points, enhancing the reliability of its results.

flake permittivity. As we found in the near-field study, both the in-plane and out-of-plane permittivity of MoS_2 is $\sim 3\%$ smaller than the permittivity from Ermolaev et al. [2], used in the reflectance fit. Therefore, if we assume the same difference of $\sim 1.5\%$ in the refractive index for the whole wavelength range and redo the reflection fit, then the fitted thickness of MoS_2 flakes will be $\sim 1.5\%$ larger than the ones shown in Table S4 (because the position of spectral minima/maxima depends strongly on the optical path length inside the flake, $n_2\, t_{MoS_2}$), agreeing even better with the results of the near-field fit. This indirectly proves the accuracy of both our near-field and far-field reflectivity fitting methods. However, one should keep in mind that in both the reflectance fit and the near-field fit the thickness and permittivity of the flakes are linked/coupled via the governing equations, while the AFM measurement depends only on the flake thickness (and on the mechanical properties of the material).

Thickness in nanometer	Flake A	Flake B	Flake C	Flake D	Flake E	Flake F
AFM measurements ±10%	82.4	185.3	250.0	325.3	355.4	458.5
Reflectance fit	81.77 ± 0.69	193.26 ± 0.21	262.50 ± 0.34	327.73 ± 0.35	416.76 ± 0.46	502.30 ± 0.57
Reflectance conformal fit	82.84 ± 0.14	192.85 ± 0.05	260.62 ± 0.12	328.02 ± 0.19	415.61 ± 0.15	503.50 ± 0.20
Near-field fit	81.5	192.7	263.3	330.6	419.3	507.3

Table S4. Thickness of each MoS_2 flake as experimentally determined from AFM, Reflectance spectroscopy, and SNOM measurements and fits.

S4. Transmission s-SNOM

The near-field measurements were performed using a customized commercially available transmission type s-SNOM (NeaSpec, Attocube). The setup (Figure S10) employs pseudo-heterodyne demodulation to simultaneously acquire amplitude and relative phase information from the near-field signal. A continuous-wave near-infrared laser beam ($\lambda_0 = 1570$ nm) is split into two paths. One path is the reference arm, where the light is modulated by an oscillating mirror (RM, $f \approx 300$ Hz). In the other path the laser beam is focused (~ 3 µm spot size) onto the edge of a flake by a parabolic mirror (PM) below the sample. A near-field probe (Pt-coated ARROW-NCPt, NanoWorld) scatters the near field into free space, transforming the bound evanescent waves into freely propagating waves.

The scattered near-field signal is collected by another PM above the sample and is further recombined with the reference beam so their interference can be detected. The detected signal is then subsequently demodulated (pseudo-heterodyne detection) at higher harmonics of the probe's oscillation frequency ($\eta\Omega$, with $\eta = 3$ and 4) to suppress the background (any light scattered from the tip or the sample, but not related to the probed near field).

When scanning, the sample is moved; therefore, to maintain the excitation beam spot at the flake edge, the bottom PM is moved synchronously with the sample.

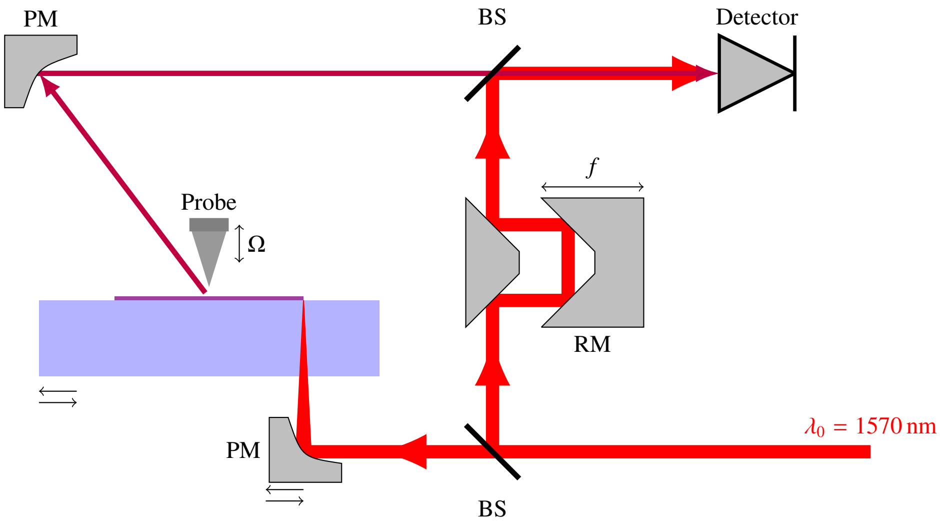

FIG. S10. Schematic illustration of the s-SNOM setup.

S5. Data processing details

Ideally, the complex-valued wavevector can be extracted directly from the Fourier spectrum. However, this requires infinitely long data, which is practically impossible. When the data is regularly sampled and of finite length L, then one can apply the classical Discrete Fourier Transform (DFT), which will have a resolution of $\Delta k = 2\pi/L$, corresponding to $\Delta N_m = \lambda/L \approx 0.04$ for our 40-µm-long scans. Additionally, DFT of our scans will result in spectral leakage because of ”windowing”, since only a 40-µm-long part of an otherwise infinitely long signal is transformed. The resolution can be improved by artificially increasing the length of the data by zeroes (so-called zero padding), but the Fourier spectrum will still be plagued by spectral leakage, resulting in spreading and overlapping of modes, which will reduce the efficiency of Fourier filtering (that is, the filtered mode in the direct space will contain a small amount of other modes, reducing the accuracy of the following fitting).

To mitigate the above issue of spectral leakage, we apply the Extended Discrete Fourier Transform (EDFT), developed by Liepins [7]. Essentially, EDFT assumes a band-limited ”true” spectrum (of unknown infinitely long data), and it iteratively extrapolates the measured data to minimize the difference between its DFT spectrum and the ”true” spectrum. For a thorough summary of EDFT see Liepins [7]. In our case, we applied EDFT with $5L$ extrapolated length (i.e., improving the resolution of DFT by 5 times) and 10 iterations (which is justified by observing no noticeable change in the Fourier spectrum after consecutive iterations). To minimize the influence of selected-by-hand parameters of the Fourier transform, we find the wavevector by the direct-space fitting of the Fourier-filtered mode.

As mentioned in the main text, our measurements suffered from not perfect synchronization between the movement of the sample and the bottom parabolic mirror during the scan due to the lower quality of the mirror stage. This resulted in a small displacement (∼ 100 nm) of the incident spot relative to the flake edge. Since the spot size is relatively large (FWHM ∼ 3 µm), this displacement has a negligible influence on the near-field amplitude, but it introduces phase wobbling. To correct the phase ‘wobbling’ and determine N_m without artificially lowering the estimated errors, we use the following two-step procedure (Figure S11):

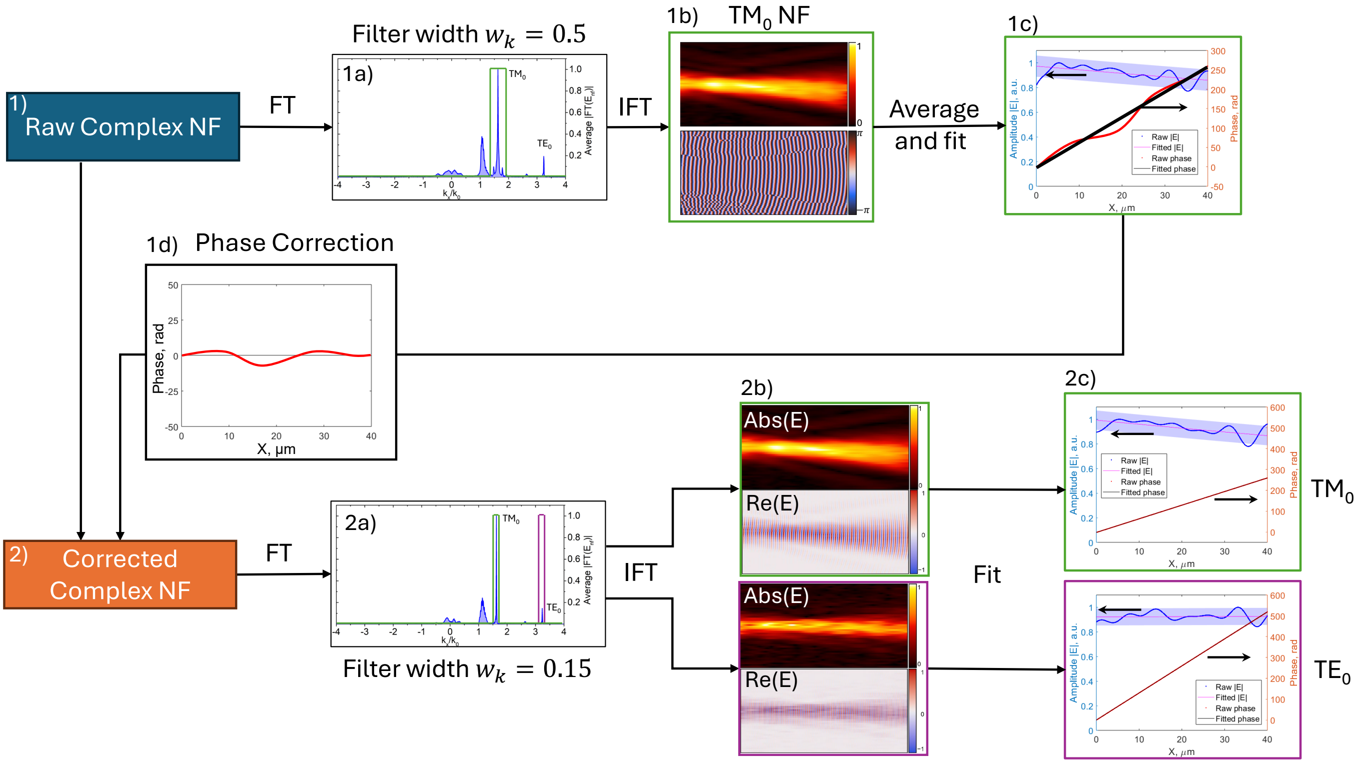

FIG. S11. Near field data processing procedure to extract the effective mode indices. **1)** The raw complex near field map is Fourier transformed (using EDFT). **1a)** Filtering of the optical modes with a square window of width $0.5k_0$. **1b)** Followed by inverse Fourier transform. **1c)** The amplitude weighted unwrapped phase is linearly fitted. **1d)** The residuals of the linear phase fit is extracted and assumed as the phase correction. **2)** The correction phase is subtracted from the raw near-field phase. **2a-c)** Similarly to steps 1a-c, the corrected near field is Fourier transformed, each mode is filtered with a square window of the width $0.15k_0$, followed by fitting in the direct space.

1) First, we select the mode with the most prominent peak in the Fourier spectrum, which does not overlap with others, and

filter it using a square window function with a width of $0.5k_0$. Then it is inversely transformed back to real space, converted to 1D by performing integration along the y-direction, followed by a linear fit of the unwrapped phase. This process provides the residual phase, which is then subtracted from the raw data.

2) In the second iteration, the corrected complex near-field data is Fourier transformed again and filtered for each mode using a smaller square window function with a width of $0.15k_0$, followed by the same procedures to provide fitted N_m. Importantly, to avoid artificial lowering of the uncertainty, we estimate the squared error of N_m for each mode as the sum of the squared error in the second step and the squared error for the reference mode in the first step.

To justify that the residual phase is indeed a phase correction for the whole data, we compare residual phases for both modes, which demonstrates strong correlation (Figure S12a). As a result, correcting the phase from the residual phase of the first mode not only reduces the residual phase of the same mode (as expected), but also reduces the residual phase of the second mode (Figure S12b). The difference in using a window width of $0.5k_0$ and $0.15k_0$, as illustrated in Figure S12b-c, does not change the estimated effective index and its error significantly. The smaller filtering window of $0.15k_0$ was chosen to separate closely spaced TE_1 and TM_0 modes of the flake C (see Figure 3 in the main text).

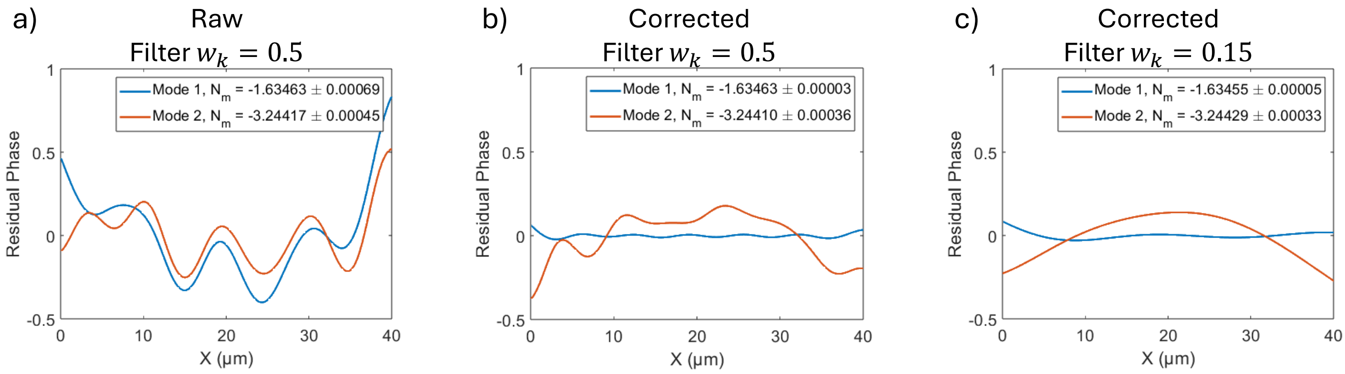

FIG. S12. Residual phase along the scan direction of **a)** the raw data filtered for the two modes, **b)** after the correction using mode 1 and filtered using a square window with width $w_k = 0.5k_0$, **c)** similarly as b) but with window width $w_k = 0.15k_0$. Mode 1 corresponds to TM_0 and mode 2 to TE_0. Figure b) and c) does not show a large difference making the use of a the small window in the corrected data filtering step acceptable.

When applying a square window function (filter), it is crucial to consider the impact of the selected window width. A window width that is too narrow may exclude valuable information, while an excessively wide window may encompass additional peaks in the spectrum, thereby reducing accuracy. In the initial step, a width of $0.5k_0$ is used to ensure a proper phase correction. However, varying this width will result in slight changes in the outcomes of the subsequent step. Figure S13 illustrates this effect for the TM_0 and TE_0 modes on the 185 nm thick flake B. It is evident that altering the initial width causes minor variations in the fitted effective mode index; however, these are negligible compared to the inherent measurement errors. Even when the width is adjusted in the second step, the results remain consistent unless the window width approaches the peak width, causing asymmetry and skewing the effective mode index. In our data processing procedure, we utilize a width of $0.15k_0$ in the second step (as mentioned above), which is the smallest feasible width that does not significantly impact the results and is still able to differentiate modes that are close in momentum space.

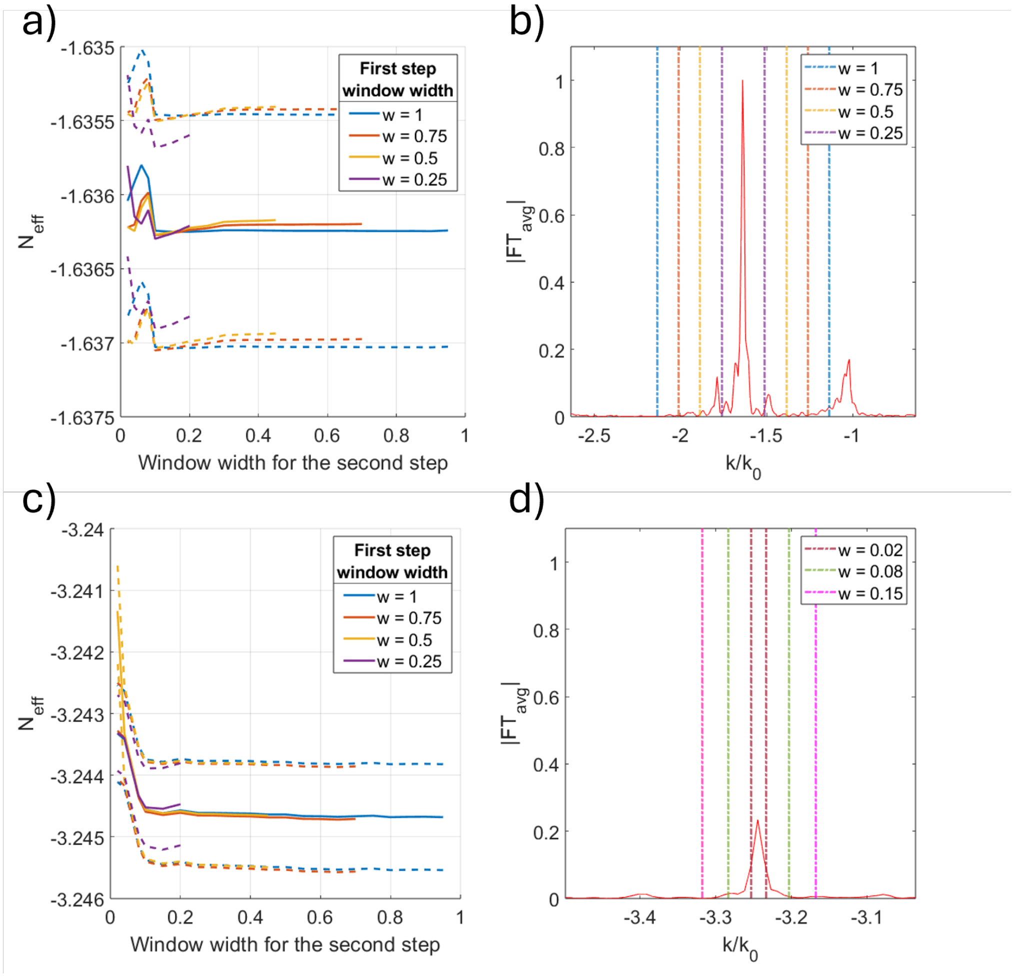

FIG. S13. **a)** and **c)** Filter convergence for the TM_0 and TE_0 modes respectively and the two steps for a measurement on the 185 nm flake (solid lines). The dashed lines indicate the error bounds. **b)** Momentum spectra (solid line) around the TM_0 mode with the filter widths (dash-dot lines) for the first step and **d)** for the second step for the TE_0 mode.

S6. Fitting the effective mode index data to extract the permittivity

There are multiple ways to determine the permittivity from the measurements, each varying in complexity and accuracy. The most straightforward approach involves using a couple of effective mode indices (one for TE and one for TM mode) measured on a flake with a thickness t and applying them directly in the dispersion relations. First, $\varepsilon_{||}$ is determined directly from the dispersion equation for TE modes (see eq. 2a the main text), which is then used in the equation for TM modes to find $\varepsilon_{\perp}$ (2b in the main text). However, this method assumes a one-way dependence between the permittivity components and considers only a single flake thickness t and a couple of effective indices. Moreover, it completely ignores the inaccuracy of t.

To account for all measurements and estimated errors, a least-squares approach is used, where estimated errors are used as weights to ensure the proper contribution from each measurement. In our approach we are looking for an unknown MoS_2 anisotropic permittivity ε^{fit} and a set of exact flake thicknesses $\mathbf{t}^{\text{fit}}$, which will minimize the following normalized deviations between measured and exact (or calculated) parameters:

$$\mathbf{F}(\varepsilon^{\text{fit}}, \mathbf{t}^{\text{fit}}) = \sum_i \left[\left(\frac{t_i^{\text{fit}} - t_i}{\Delta t_i} \right)^2 + \sum_m \left(\frac{N(\varepsilon^{\text{fit}}, t_i^{\text{fit}}) - N_{i,m}}{\Delta N_{i,m}} \right)^2 \right], \tag{S17}$$

where the summation goes over all flakes (labeled with index i) and all supported TE and TM modes (labeled with index m), whose effective mode indices are calculated from ε and $\mathbf{t}^{\text{fit}}$ by using the dispersion equations.

The above least-squares approach can be solved directly by searching all unknown 8 parameters at once (2 parameters of ε and 6 parameters of t^{fit} for flakes A-F). However, such fitting is relatively heavy and might be unstable, therefore we decided to apply the following nested fitting:

1) If ε is known, then the thickness of each flake t_i^{fit} can be determined by the least-squares fitting, minimizing the following corresponding deviation:

$$\mathbf{F_i}(t_i^{\text{fit}}) = \left(\frac{t_i^{\text{fit}} - t_i}{\Delta t_i} \right)^2 + \sum_m \left(\frac{N(\varepsilon, t_i^{\text{fit}}) - N_{i,m}}{\Delta N_{i,m}} \right)^2, \tag{S18}$$

which implicitly defines thickness as a function of ε, $t_i^{\text{fit}}(\varepsilon)$. Unlike in the straightforward approach, here it is only one fitting parameter t_i^{fit}.

2) The above implicit definition of t_i^{fit} is used in Eq. S17, leaving only 2 fitting parameters: $\varepsilon_{||}$ and $\varepsilon_{\perp}$.

Thus, our nested fitting procedure implicitly defines the permittivity and flake thicknesses as a function of measured parameters:

$$(\varepsilon^{\text{fit}}, \mathbf{t}^{\text{fit}}) = Q(\mathbf{t}, \mathbf{N}, \Delta\mathbf{t}, \Delta\mathbf{N}). \tag{S19}$$

We have used a function 'fsolve' in MATLAB software to find effective mode indices from dispersion equations and do the least-squares fitting. The initial guess for the fitting parameters are the corresponding flake thicknesses measured by AFM and the permittivity taken from Ermolaev et al. [2] ($\varepsilon_{||} = 16.56$, $\varepsilon_{\perp} = 6.43$ at our free-space wavelength of 1570 nm).

To estimate the uncertainty of our fitted parameters, we have assumed all our measurements being independent (resulting in the upper bound estimation of the uncertainty). By using standard sensitivity analysis, we estimate the error contribution of each measured value as its derivative, multiplied by the corresponding error. Then all these contributions are squared and added to estimate the uncertainty of the permittivity:

$$\Delta\varepsilon = \sqrt{\sum_i \left[\left(\frac{\partial Q}{\partial t_i} \Delta t_i \right)^2 + \sum_m \left(\frac{\partial Q}{\partial N_{i,m}} \Delta N_{i,m} \right)^2 \right]}. \tag{S20}$$

The partial derivatives of Q are obtained simply by a finite difference approximation:

$$\frac{\partial Q}{\partial t_j} = \frac{Q\big(\mathbf{t}(t_j + \Delta_t), \mathbf{N}\big) - Q(\mathbf{t}, \mathbf{N})}{\Delta_t}, \tag{S21}$$

$$\frac{\partial Q}{\partial N_{j,i}} = \frac{Q\big(\mathbf{t}, \mathbf{N}(N_{j,i} + \Delta_N)\big) - Q(\mathbf{t}, \mathbf{N})}{\Delta_N}, \tag{S22}$$

where Δ is a small perturbation. The changes in Δ_t and Δ_N are consistent across all derivatives and should be selected to be as small as possible, while still being sufficiently large to avoid issues related to numerical precision. We chose $\Delta_t = 1$ nm and $\Delta_N = 0.001$, which corresponds approximately to 10% of the smallest error in $\Delta\mathbf{t}$ and $\Delta\mathbf{N}$.

References

[1] SCHOTT, "Optical glass datasheets," (N-BK7 data as of 01-Dec-2023).

[2] G. A. Ermolaev, D. V. Grudinin, Y. V. Stebunov, K. V. Voronin, V. G. Kravets, J. Duan, A. B. Mazitov, G. I. Tselikov, A. Bylinkin, D. I. Yakubovsky, S. M. Novikov, D. G. Baranov, A. Y. Nikitin, I. A. Kruglov, T. Shegai, P. Alonso-González, A. N. Grigorenko, A. V. Arsenin, K. S. Novoselov, and V. S. Volkov, Nat. Commun. **12**, 854 (2021).

[3] B. Munkhbat, P. Wróbel, T. J. Antosiewicz, and T. O. Shegai, ACS Photonics **9**, 2398 (2022).

[4] P. G. Zotev, Y. Wang, D. Andres-Penares, T. Severs-Millard, S. Randerson, X. Hu, L. Sortino, C. Louca, M. Brotons-Gisbert, T. Huq, S. Vezzoli, R. Sapienza, T. F. Krauss, B. D. Gerardot, and A. I. Tartakovskii, Laser Photon. Rev. **17**, 2200957 (2023).

[5] B. R. Carvalho, L. M. Malard, J. M. Alves, C. Fantini, and M. A. Pimenta, Phys. Rev. Lett. **114**, 136403 (2015).

[6] Y. Fang, J. Pan, J. He, R. Luo, D. Wang, X. Che, K. Bu, W. Zhao, P. Liu, G. Mu, H. Zhang, T. Lin, and F. Huang, Angewandte Chemie International Edition **57**, 1232 (2018).

[7] V. Liepins, arXiv **1303.2033v15** (2024), 10.48550/arXiv.1303.2033.
